# Rural context, single institution prospective outcomes after enhanced recovery colorectal surgery protocol implementation

**DOI:** 10.1186/s12913-020-05971-3

**Published:** 2020-12-03

**Authors:** Levi Smucker, Jennifer Victory, Melissa Scribani, Luis Oceguera, Raul Monzon

**Affiliations:** Bassett Medical Center, Cooperstown, NY USA

**Keywords:** Enhanced recovery, ERAS, Colorectal surgery, Rural, Organizational change

## Abstract

**Background:**

Rural hospitals face unique challenges to adopting Enhanced Recovery protocols after colorectal surgical procedures. There are few examples of successful implementation in the United States, and fewer yet of prospective, outcomes-based trials.

**Methods:**

This study drew data from elective bowel resection prospectively collected, retrospectively analyzed cases 2 years prior (*n* = 214) and 3 years after (*n* = 224) implementing an ERAS protocol at a small, rural health network in upstate New York. Primary outcomes were cost, length-of-stay, readmission rate, and complications.

**Results:**

The implementation required changes and buy-in at multiple levels of the institution. There was a statistically significant reduction in mean length of stay (6.9 versus 5.1 days) and per-patient savings to hospital ($3000) after implementation of ERAS protocol. There was no significant change in rate of 30-day readmissions or complications.

**Conclusions:**

The authors conclude that for rural-specific barriers to implementation of Enhanced Recovery protocols there are specific organizational strategies that can ultimately yield sustainable endpoints.

## Background

Rural hospitals face relative challenges in the adoption of Enhanced Recovery After Surgery (ERAS) protocols for their patients. When the ERAS Study Group published consensus guidelines in 2005, the protocols had primarily been developed and used in urban and academic centers in Europe [1]. Eventually, the ERAS Society --- established in 2010 --- began to create accessible education materials and audit systems to disseminate and encourage early adoption of best perioperative practices throughout the world [2]. Early adoption in North America began soon thereafter. Since then, ERAS protocols have ballooned to encompass multiple surgical specialties beyond colorectal surgery, and can refer to many forms of multi-modal, comprehensive, peri-operative frameworks. ERAS protocols rely on a multitude of practices which invariably include detailed preoperative education and counseling, medical optimization, tight glycemic control, maintenance of normothermia, multimodal analgesia, opioid reduction, early feeding, early mobilization, and early catheter removal.

However, academic and urban hospitals have implemented ERAS at much greater rates than rural hospitals. There are unique challenges to ERAS feasibility in rural practice including patient factors, geographic limitations, high staff turnover and shortages, fewer resources, and lower case volume. Some other barriers cited include patient education and the notion that ERAS principles may not be intuitive [3, 4].

There is motivation to start an ERAS protocol despite rural colorectal surgery challenges. There is significant meta-data to demonstrate both improved outcomes [5] and cost-savings [6] in ERAS implementation, even in resource-poor hospitals [7]. However, there is paucity of outcomes research data for ERAS in rural context. Indeed, a comprehensive, broad-term literature search of the results of Enhanced Recovery in colorectal surgery in a rural and context yields only 1 published example of North American rural ERAS prospective data [8], and several examples of such research in European rural [9–11], or North American urban community hospital networks [12, 13]. Please refer to Additional file 1: Appendix 1. Therefore, the objective of the study was to determine the feasibility of ERAS implementation intervention in the rural context, by *tracking and describing* the organizational adoption of the protocol in addition to measuring Length-of-stay, cost, and complication rates.

## Methods

The author’s rural context is the Bassett Healthcare Network (BHN), which is a regional health care system serving central New York State, including 9 counties, containing 5 critical access hospitals, and 1 central hospital of 126 beds which is a teaching hospital. The BHN’s residents are, on average, 98.6% rural, as defined by United States Census Bureau Data. The median annual household income was $53,079, with 11.3% of families living below poverty in the region. One-third of adults older than 25 had attained an associate’s degree or higher. In Otsego County, the center of BHN, 94% of residents identify as white alone, 21.7% are over the age of 65.

We drew data from elective bowel resection cases 2 years prior (*n* = 214) and 3 years after (*n* = 224) implementing an ERAS protocol at a small, rural health network in upstate New York. All patients undergoing elective, non-emergent bowel resection within the study timeframe were eligible and de-identified. Cases were identified by 2 individuals querying all operative electronic medical records in the study timeframe by CPT (Current Procedural Terminology) code search for bowel-resection procedures, and emergent cases were excluded. Total study size was obtained post-intervention after accrual of patients equaled eligible patients, pre-intervention. The implementation required changes in staff workflow, protocolized order set design, buy-in at multiple levels of perioperative care, and at least weekly or biweekly meetings of multidisciplinary teams throughout 6 months prior to “roll-out”. Departments involved included Surgery, Anesthesiology, Nutrition, Case Management, Physical Therapy, Information Technology, and Management/Administration. Strategies for implementation are seen in Table 3. While our institution-specific ERAS order sets and protocols---based on ERAS Society guidelines---are defined in Additional file 1: Appendix 2, the following interventions were most relevant: Usage of carbohydrate loading; Multimodal pain management (including liposomal bupivacaine); Avoidance of routine intra/post-operative nasogastric decompression; Early removal of urinary catheters; Early cessation of intravenous fluids postoperatively; Usage of Alvimopan; Early regular diet and ambulation.

Pre versus post differences in Length-of-stay (LOS) and surgical time were tested using the independent samples t test. Comparisons between patient characteristics in pre and post conditions were done by chi-square for categorical variables (eg gender, presence of comorbidities) or by the independent samples t test for continuous variables (eg age). All statistical analyses were carried out using SAS version 9.3. A two-way analysis of variance (ANOVA) model was constructed to test for a differential change in length of stay pre and post-ERAS for laparoscopic versus open procedures. For this analysis, the F test of interest was for the interaction term of procedure type by time. The cost savings was calculated to be approximately $3000 by extrapolating the average cost incurred to hospital per day, multiplied by reduction in LOS.

## Results

A total of 438 bowel resection cases were included in this analysis; 214 resections prior to implementing the ERAS protocol and 224 resections after the protocol. Data and measurements were obtained from electronic medical record documentation. The average age for all subjects was 60.9 years (standard deviation = 14.6 years); 50.7% subjects were female and 49.3% were male. There were no statistically significant differences in patient characteristics pre-protocol versus post-protocol. A greater proportion of procedures were performed laparoscopically after implementation of the ERAS protocol (*p* < 0.0001). The distribution of patient characteristics and case types pre- and post-ERAS are shown in Table 1. Our compliance rate---after 1 month of implementation---for usage of pre-op *and* post-op order sets was 75.89%, while the rate for usage of pre-op *or* post-op order sets was 93.75%.

**Table 1:** Patient Characteristics, Type of Resection, Pre- and Post- Intervention

		Pre-ERAS (*n* = 214)	Post-ERAS (*n* = 224)	*p*-value
Patient Characteristics	Age, mean (SD)	60.7 (15.2)	61.2 (14.1)	*p* = 0.73
	Male, n (%)	112 (52.3)	104 (46.4)	*p* = 0.22
	Female, n (%)	102 (47.7)	120 (53.6)	
	BMI (mean, SD	29.7 (7.5)	30.2 (7.0)	*p* = 0.50
	ASA Class 1, n (%)	0	2 (0.9)	*p* = 0.33
	ASA Class 2	94 (43.9)	96 (42.9)	
	ASA Class 3	106 (49.5)	117 (52.2)	
	ASA Class 4	14 (6.5)	9 (4.0)	
	Diabetes, n (%)	47 (22.0)	51 (22.8)	*p* = 0.84
	CAD, n (%)	31 (14.5)	32 (14.3)	*p* = 0.95
	Current Smoker n (%)	39 (18.2)	49 (21.9)	*p* = 0.34
	COPD, n (%)	24 (11.2)	32 (14.3)	*p* = 0.34
	CHF, n (%)	16 (7.5)	12 (5.4)	*p* = 0.36
	History of Multiple Cancers, n (%)	12 (5.6)	13 (5.8)	*p* = 0.93
	History of IBD, n (%)	16 (7.5)	22 (9.8)	*p* = 0.38
Type of Resection	Small Bowel, n (%)	21 (9.9)	25 (11.2)	*p* = 0.02
	Ileocecectomy	12 (5.6)	21 (9.4)	
	Right Colon	64 (30.1)	57 (25.6)	
	Extended Right Colon	4 (1.9)	57 (25.6)	
	Left Colon	7 (3.3)	13 (5.8)	
	Sigmoid	39 (18.3)	59 (26.5)	
	LAR	57 (26.8)	42 (18.8)	
	APR	0	3 (1.4)	
	Total Colectomy	6 (2.8)	1 (0.5)	
	Total Proctocolectomy	3 (1.4)	1 (0.5)	
	Open, n (%)	49 (22.9)	18 (8.0)	< 0.0001
	Laparoscopic	57 (26.6)	111 (49.6)	
	Hand-assisted	88 (41.1)	86 (38.4)	
	Combo Lap/open	20 (9.4)	9 (4.0)	

There was a statistically significant reduction in length of stay and associated total admission time after implementation of the ERAS protocol (Table 2). There were no differences in readmission rate or statistically significant overall complication rates pre versus post implementation. The prevalence of any complication after surgery did not differ pre versus post ERAS, even when stratified by specific complication. Rates of specific complications are shown in Additional file 1: Appendix 3.

**Table 2:** Measured Outcomes Pre- and Post- Intervention

	Pre-ERAS	Post-ERAS	*p*-value
Mean LOS (Days)	6.9	5.1	*p* < 0.0001
Open (Days)	10.3	7.2	**
Laparoscopic (Days)	4.9	3.9	**
Mean Total Admission Time (Days)	6.88	4.3	*P* < 0.0001
Mean Surgical Time (Hours)	4.1	3.9	*p* < 0.22
Readmitted to Hospital Within 30 days (% of cases)	16.6	15.7	*p* = 0.80
Any Complication (% of cases)	29.4	34.8	*p* = 0.23

**p(interaction) = 0.13

**Fig. 1:**
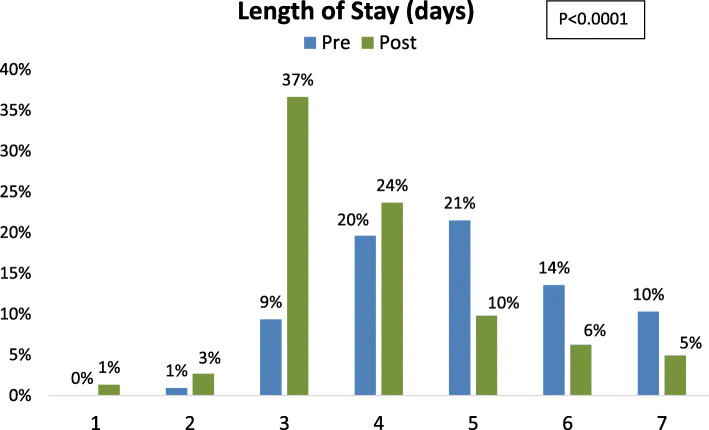
Percent of Patients Demonstrating Given Length of Stay

Considering subjects with a length of stay of a week or less (Fig. 1), a clear shift was observed in the distribution of length of stay post-ERAS. In the post condition, the most frequent length of stay was 3 days, and more than two-thirds of patients were discharged on day four or sooner. In the pre condition, only one-third of patients were discharged at or before day four.

## Discussion

In this study, we prospectively collected and retrospectively analyzed bowel resection patients in a single institution both before and after implementation of an ERAS protocol. The volume of resections is relatively large for international rural standards, but this volume was attainable through the large geographic catchment area. Overall, the greatest effect of the Enhanced Recovery interventions was observed in decreased length of stay of 1.8 days on average. This resulted in a net savings of approximately $3000 per patient at our institution. Notably, there was no statistically significant difference in the rates of any complication nor readmissions pre and post ERAS. Additionally, rates of commonly reported colorectal complications are commensurate with nationally reported rates [14, 15]. It is unclear why our institution did not see an improvement in complication rates as demonstrated in other meta-analyses of ERAS trials [16, 17]. However, we did not see an increase in readmission rates either, which is frequently reported in other studies [18].

The study was not randomized, and this is a limitation. The intervention (ie/ ERAS protocol) was not blinded, and this is a source of potential bias. However, patient groups pre and post were comparable in demographics and comorbidities. It was necessary to maximize the number of patients subjected to protocol in a short period, and this required a total overhaul in the institutional practice. The compliance rates of order sets (75–93%) are better than, or commensurate with, other published rates [18, 19].

One of the most important components was the support of the organization, which permitted the needed devoted meetings with all representatives of pre, trans, and post operative levels of care. Every relevant party was included. The changes hoped for were discussed, and there was consensus. These interventions started in the clinic setting at the network level, and flowed through the perioperative period. The unique, complex nature of implementing these changes may also represent a limitation of generalizability to other organizations.

At the core, the sustainable endpoint of any project implementation requires shifting culture by convincing those involved of the intrinsic benefits. From a patient’s perspective, this may include convincing a patient that carbohydrate loading, early ambulation, and a clear recovery timeline will be best for them. This was accomplished through clear, lay-term pamphlets in clinic and prominent ERAS-educational whiteboards in patient rooms. From a health caregiver-provider perspective, this included in-service training providing convincing data that their patient’s would benefit from the interventions.

We believe this demonstrates that ERAS is feasible for rural hospitals, despite unique challenges in the rural setting. Lack of human labor, poor communication and collaboration, resistance to change, rotating residents, and patient factors (such as comorbidity and socioeconomic disadvantages) have all been listed as barriers in other publications [20, 21]. Table 3 demonstrates how a “rural-specific barrier” to implementation can be overcome, yielding a sustainable endpoint. Several of these sustainable endpoints are difficult to quantify, such as trusted relationships or lessened burden of opioid addiction on the community. There is opportunity for more research therein.

**Table 3:** Implementation Model for ERAS Protocol Barriers

Rural-Specific Barrier	Implementation	Sustainable Endpoint
Patient’s travel distance to hospital	Build “hub and spoke” model hospital network	Surgery performed at larger hospital but patients are seen closer to home for pre- and post-operative visits
Poor patient health literacy	Design communicable pre- and post-operative counseling (pamphlets and posters created, in lay terms) and offer easy access for communication via telephone or internet	Trusted relationships develop between patients and providers
Relatively low-volume surgical practice	Use evidence-based changes in practice	Improved outcomes yield change of culture
Care staff education challenges in the face of workforce shortages and high turnover	Streamline processes, standardized order sets, educate staff about the benefits of ERAS	Measurable goals are transparent for all. Intrinsic motivation of caregivers that ERAS is best for patients. Reduce total patient-days on wards.
Few financial resources for equipment and medication, higher percent Medicare and Medicaid patients, lower reimbursement	Implement accelerated post-operative track with safe discharge. Prioritize stock of ERAS components, multimodal analgesia and justify to payers and administrators	Cost-containment through lower LOS, complications and readmission

## Conclusion

In a single-institution rural hospital, implementation of ERAS protocol yielded significantly decreased length-of-stay, without effect in complication rate or readmission rate. Enhanced Recovery protocols, therefore may offer significant value for rural medical systems, despite inherent challenges in their implementation.

## Supplementary Information

**Additional file 1.**

## Data Availability

The availability of supporting data exists proprietary to the Bassett Research Institute and no other repositories public nor private were used. Since the impetus for this research was as internal quality improvement metrics, the raw data is not intended for sharing at this time, a decision made by the Bassett Research Institute.

## Abbreviations

ERAS: Enhanced Recovery After Surgery
BHN: Bassett Healthcare Network
LOS: Length-of-stay
CPT: Current Procedural Terminology
