# Supplementary material for: Rural context, single institution prospective outcomes after enhanced recovery colorectal surgery protocol implementation
Source: BMC Health Serv Res. 2020 Dec 3;20:1120. doi: 10.1186/s12913-020-05971-3 (PMC7712524; doi:10.1186/s12913-020-05971-3)
Supplement: Supplementary file 1 — Additional file 1. [file 12913_2020_5971_MOESM1_ESM.docx]

**+ Appendix 1:**

Boolean search strategies of rural ERAS literature:

(Enhanced Recovery After Surgery) AND

(Colorectal) AND

(Cost)

=64 results

(Enhanced Recovery After Surgery) AND

(Colorectal) AND

(Rural)

=3 results

(Enhanced Recovery After Surgery) AND

(Colorectal) AND

(Community)

=21 results

These results were reviewed to assess if they were trials/studies, relating to ERAS, and conducted in rural centers.

**++ Appendix 2:**

Pre Admission Testing Clinic

Laboratory tests (those indicated): Protime-INR, APTT, CBC, BMP, Hgb A1C, AST, ALT, Alkaline Phosphatase, Gamma GT, Total Bilirubin, Albumin, TSH, Urine Pregnancy, Urinalysis, Urine Culture, Type and Screen

Cardiac: EKG 12-lead, Echocardiogram Transthoracic (if indicated), Standard Treadmill Test (if indicated), Nuclear Medicine Scan (if indicated)

Pulmonary (if indicated): Pulmonary Function Test

Imaging: CT Abdomen Pelvis with and/or without IV contrast with PO contrast, US abdomen limited, Chest X-Ray

Ambulatory referral to any of the following: Ostomy Care, Nutrition Services, Cardiology, Pulmonology, Palliative Care, Pain Clinic, Social Work

Diet and Nutrition: High Protein Supplemental Shake

Day Prior to Surgery

Preparations: Chlorhexidine Scrub.  Mupirocin nasal ointment. Magnesium Citrate vs Miralax vs Polyethylene Glycol vs Ducolax tablet vs Metronidazole tablet vs Neomycin tablet

Day of Surgery

Laboratory tests (those indicated): Protime-INR, APTT, CBC, Creatinine, Electrolyte Panel, Glucose Check, Urinalysis, Urine Pregnancy, Urine Culture, Type and Screen vs Type and Cross,

Incentive Spirometry, 500mL Bolus Lactated Ringers, 12 mg capsule Alvimopan, Cefazolin 2mg vs 3mg and Metronidazole (No Beta-Lactam Allergy) vs Ampicillin-Sulbactam (No Beta-Lactam Allergy) vs Cetriaxone and Metronidazole ( No Beta-Lactam Allergy) vs Levofloxacin and Metronidazole (Beta-Lactam Allergy) vs Vancomycin and Metronidazole (Beta-Lactam Allergy) vs Clindamycin (Beta-Lactam Allergy)

Intraoperative Orders

Place Intermittent Pneumatic Compression Device, Bacitracin/Polymyxin sodium chloride irrigation

Analgesics: Intravenous Acetaminophen, Ketorolac, Gabapentin, Liposomal Bupivacaine

Postoperative Orders

Admission to surgical floor

Code Status selection

Vital signs every 8 hours

Ambulate 4 times Daily and out of bed to chair for at least 6 hours a day

Remove Urinary Catheter at 0500 AM of POD1

Patient may shower

Provide chewing gum to patient

Regular Diet vs Other diets indicated

Physical Therapy

Occupational Therapy

Dietician/Nutrition consultation

Social Work, Palliative Care, Pain Management, Smoking cessation, wound and ostomy consultations (if indicated)

Respiratory evaluations, inhalers, adjuncts (if indicated)

Determination of Venous Thromboembolism risk level and selection of sequential compression devices and/or subcutaneous heparin and/or enoxaparin when indicated

Daily Laboratory Tests (those indicated): Type and Screen, Electrolyte Panel, BUN, Creatinine, Glucose, Ionized Calcium, Magnesium, Troponin I, Lactic Acid, CBC with autodifferential

Alvimopan 12 mg oral two times daily

Ondansetron oral or IV as needed

Promethazine oral or IV or suppository as needed

 Cefazolin 2mg vs 3mg and Metronidazole (No Beta-Lactam Allergy) vs Ampicillin-Sulbactam (No Beta-Lactam Allergy) vs Cetriaxone and Metronidazole ( No Beta-Lactam Allergy) vs Levofloxacin and Metronidazole (Beta-Lactam Allergy) vs Vancomycin and Metronidazole (Beta-Lactam Allergy) vs Clindamycin (Beta-Lactam Allergy)

As needed opioid medications: Hydrocodone-Acetaminophen tablet vs Oxycodone tablet vs Morphine tablet vs Morphine Injectable vs Fentanyl Injectable vs Patient Controlled Analgesia (Morphine vs Fentanyl vs Hydromorphone)

Naloxone injection as needed for opioid reversal

Bowel Regimen (if indicated) of Docusate and Senna and Polyethylene Glycol and/or suppository

Smoking Cessation

Nicotine patches and/or Nicotine gum (if indicated, dosage based on number of cigarettes per day)

Insulin (if indicated)

Multivitamin tablet oral daily

+++ **Appendix 3**

| Type of Complication | Pre-ERAS (% of cases) | Post-ERAS (% of cases) | p-value |
| --- | --- | --- | --- |
| UTI | 4 | 6 | All p-values >0.05 |
| Stroke | 0 | 0 |  |
| Deep Wound Infection | 3 | 3 |  |
| Superficial Wound Infection | 6 | 9 |  |
| Pneumonia | 3 | 3 |  |
| Leak | 2 | 2 |  |
| DVT | 0 | 1 |  |
| AKI | 6 | 9 |  |
| Death | 2 | 2 |  |
| Ileus | 8 | 11 |  |
| Return to OR | 4 | 5 |  |
| CDiff | 4 | 2 |  |
